# Targeted mutagenesis and high-throughput screening of diversified gene and promoter libraries for isolating gain-of-function mutations

**DOI:** 10.3389/fbioe.2023.1202388

**Published:** 2023-07-17

**Authors:** Herbert M. Huttanus, Ellin-Kristina H. Triola, Jeanette C. Velasquez-Guzman, Sang-Min Shin, Rommel S. Granja-Travez, Anmoldeep Singh, Taraka Dale, Ramesh K. Jha

**Affiliations:** ^1^ Bioscience Division, Los Alamos National Laboratory, Los Alamos, NM, United States; ^2^ Agile BioFoundry, Emeryville, CA, United States; ^3^ BOTTLE Consortium, Golden, CO, United States

**Keywords:** protein engineering, synthetic biology, promoter engineering, mutagenesis, polymerase chain reaction, overlap extension PCR, fluorescence-activated cell sorting, whole-cell biosensor

## Abstract

Targeted mutagenesis of a promoter or gene is essential for attaining new functions in microbial and protein engineering efforts. In the burgeoning field of synthetic biology, heterologous genes are expressed in new host organisms. Similarly, natural or designed proteins are mutagenized at targeted positions and screened for gain-of-function mutations. Here, we describe methods to attain complete randomization or controlled mutations in promoters or genes. Combinatorial libraries of one hundred thousands to tens of millions of variants can be created using commercially synthesized oligonucleotides, simply by performing two rounds of polymerase chain reactions. With a suitably engineered reporter in a whole cell, these libraries can be screened rapidly by performing fluorescence-activated cell sorting (FACS). Within a few rounds of positive and negative sorting based on the response from the reporter, the library can rapidly converge to a few optimal or extremely rare variants with desired phenotypes. Library construction, transformation and sequence verification takes 6–9 days and requires only basic molecular biology lab experience. Screening the library by FACS takes 3–5 days and requires training for the specific cytometer used. Further steps after sorting, including colony picking, sequencing, verification, and characterization of individual clones may take longer, depending on number of clones and required experiments.

## 1 Introduction

In the field of synthetic biology, rational design of proteins and promoters has gained extensive interest, especially for metabolic engineering efforts (Blazeck and Alper, 2013; Xiong et al., 2021). For proteins, this is frequently achieved by site directed mutagenesis of specific codons in the genes (Alberghina and Lotti, 2005). For promoter engineering, the method is less streamlined, but commonly randomization of ribosomal binding sites (RBS) is a preferred method for tuning the expression of genes (Salis et al., 2009; Zhang et al., 2015; Oesterle et al., 2017; Segall-Shapiro et al., 2018). Targeted mutagenesis is an essential method for achieving gain-of-function mutations in a gene or promoter. However, introducing single mutations one at a time and individually testing for changes in function is a tedious and time-intensive process, which at best only results in incremental changes to phenotype after each mutation. The protocol described herein eliminates the bottleneck of individually testing variants of genes and promoters (generated by site-directed mutagenesis) by assaying the combinatorial effect of several mutations at once, as part of a large multi-variant library.

**FIGURE 1:**
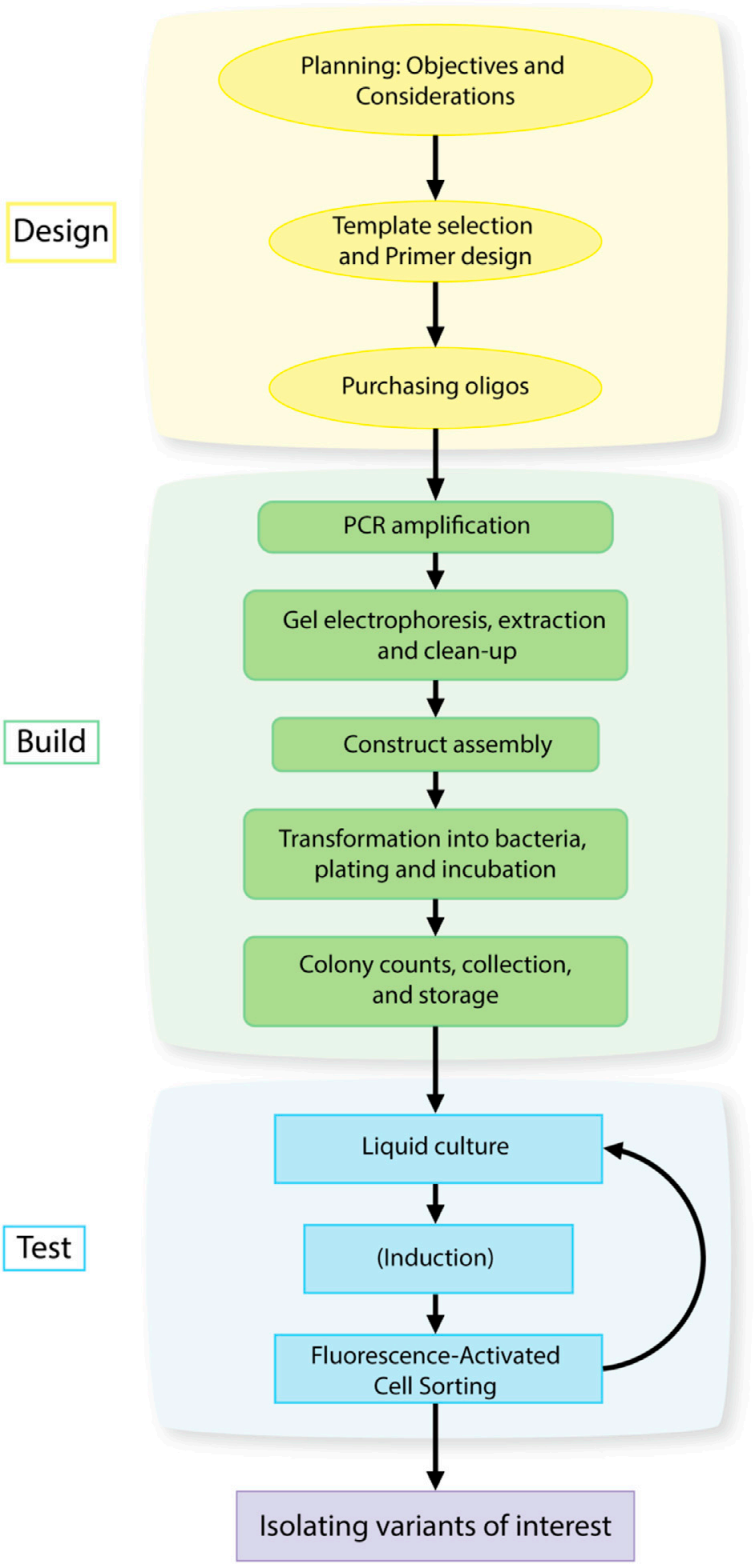
Design-Build-Test workflow for isolating gain-of-function mutations in a gene or promoter using library generation and high-throughput screening.

The method described here uses a combination of overlap extension Polymerase Chain Reaction (PCR) (Ho et al., 1989; Horton et al., 1989) and saturation or partial saturation mutagenesis with degenerate primers (Kretz et al., 2004) to produce a library of gene and promoter variants that can then be screened for desired characteristics (outlined in Figure 1). Oligonucleotide overlap extension with degenerate codons is a simple yet powerful technique to introduce massive numbers of mutations, while using only a relatively simple two-step PCR. Economically, it takes advantage of the low cost for oligonucleotides (<70 bp), and the possibility of introducing degeneracy in a desired region. These oligos can then be used in a two-step PCR (fragment generation followed by assembly of fragments) to rapidly generate libraries with diversity on the order of 10⁴–10⁷ variants. The resulting libraries lend themselves to high-throughput screening via fluorescence activated cell sorting (FACS), when coupled to a relevant fluorescent reporter, allowing for the rapid identification and isolation of variants of interest, which can then be individually characterized.

For the successful application of this method, a variety of factors need to be taken into account when designing a library. The following sections outline important considerations when aiming to design, construct and screen a promoter or a protein library (Sections 1.1, 1.2 respectively).

### 1.1 Promoter and RBS libraries

Promoters may need to be modified for a variety of reasons including, tuning gene expression (Bakke et al., 2009; Boldrin et al., 2017), pathway optimization (Jin et al., 2019), designing synthetic circuits (Xie and Fussenegger, 2018), engineering biosensors (Pardo et al., 2020; Bentley et al., 2020) and providing new basic molecular biology tools for non-model organisms (Mordaka and Heap, 2018). While many of the mechanisms linking promoter sequence to transcription rates are known and several bioinformatic tools (Cassiano and Silva-Rocha, 2020; LaFleur et al., 2022) can help predict promoter strength from sequence alone, it is still necessary to test engineered promoters experimentally. The need for combinatorial building and testing of promoter regions and sequences in its proximity is commonly the method of choice. Thus, many attempts to modify promoter performance rely on semi-rational mutation libraries. In this context, semi-rational refers to the approach of targeting specific regions of the promoter, known to be involved with various mechanisms of transcription or translation, rather than just randomly mutating the entire promoter region.

For transcription, the regions approximately 35 and 10 bases upstream of the transcriptional initiation site (referred to as −35/−10 promoter sites) are particularly important for transcription initiation and therefore a single nucleotide mutation here can have dramatic effects (Einav and Phillips, 2019). Sometimes, if the goal is to create more subtle changes in translation rate or a series of promoters with steadily increasing or decreasing strength, it may be beneficial to mutate nearby areas, but leave the −35 and −10 regions unmutated (Mordaka and Heap, 2018). When importing a promoter from one organism into another host, however, it becomes imperative to also change the −35/−10 sites. This strategy has contributed to achieving gain-of-function, allowing novel biosensors to be created in new host organisms such as *Pseudomonas putida* (Bentley et al., 2020), *Acinetobacter baylyi* ADP1 (Pardo et al., 2020) and *Corynebacterium glutamicum (Velasquez-Guzman and Huttanus et al, unpublished data).*

Regions around or within the −35/−10 sites can also contain operator regions for transcription factors. These operator regions are usually palindromic or pseudo-palindromic sequences to which the DNA binding domains of the transcription regulator bind. There are usually anywhere from one to three such sequences around the promoter region. Modification of the operator region can result in modulated binding affinity of the transcription regulator to the operator region and hence altered function. Randomization of only a few nucleotides in the operator region showed a wide range of repression levels of LacI that included increase in amplitude of response, very tight repression, or very weak repression resulting in constitutive activity of the promoter (Maity et al., 2012).

In addition to adjusting transcription rates via promoter and operator sites, further gene expression can be controlled at the translational level. While not technically part of the promoter, the ribosome binding site or RBS is typically located just between the promoter and the gene to be regulated, allowing for modifications to the RBS to be conveniently included in the promotor library. Mutation libraries of the RBS have been used to modulate and optimize translation rates in a variety of applications (Oesterle et al., 2017). Similarly, *cis*-acting elements on the mRNA can have a profound effect on translation rates (Gebauer et al., 2012; Rhodius et al., 2012) and have also been diversified to generate libraries with a wide range of expression levels of downstream genes (White, 2015; Pandey et al., 2022).

Through these and other methods, many sets of constitutive promoters covering a wide range of transcription rates have been developed. Yet there is still a need to engineer promoter sets for microbial hosts that include living therapeutics and other microbiota (Waller et al., 2017; Charbonneau et al., 2020; Dosoky et al., 2020), as well as non-model host strains for biomanufacturing purposes. A need for tuning constitutive promoters arises especially when the gene product shows instability in function or is toxic to the microbial host. In such cases, randomization of specific regions in the promoter such as −35/−10 sites, can tune down the constitutive promoter, resulting in stable expression of the downstream gene. The approach was successfully applied in tuning down the expression of *mucK* transporter gene for stable expression from a constitutive promoter in *Pseudomonas putida* (Shin et al., 2022).

Inducible promoters are an important component of the molecular biology toolkit (Chen et al., 2018), as they provide timely or dynamic regulation, and are often included in gene circuits (Mayo et al., 2006; Xie and Fussenegger, 2018) and metabolic pathway optimization (Jin et al., 2019). When engineering a synthetic inducible promoter, important aspects to consider include the inducibility and background activity of a promoter. Induction should be relatively straightforward for most cases, not requiring expensive chemicals, specific growth media or temperature shifts. In addition, an inducible promoter should show distinct activity when the inducer is present and low background activity when the inducer is absent. It is important to have tight regulation to avoid the basal expression levels interfering with the interpretation of the results (Hartman et al., 2011). Engineering these desirable qualities into a promoter, requires knowledge of the induction mechanism for targeted mutagenesis and, sometimes in addition, a semi-rational design of a library from which the desirable response can be isolated.

**FIGURE 2:**
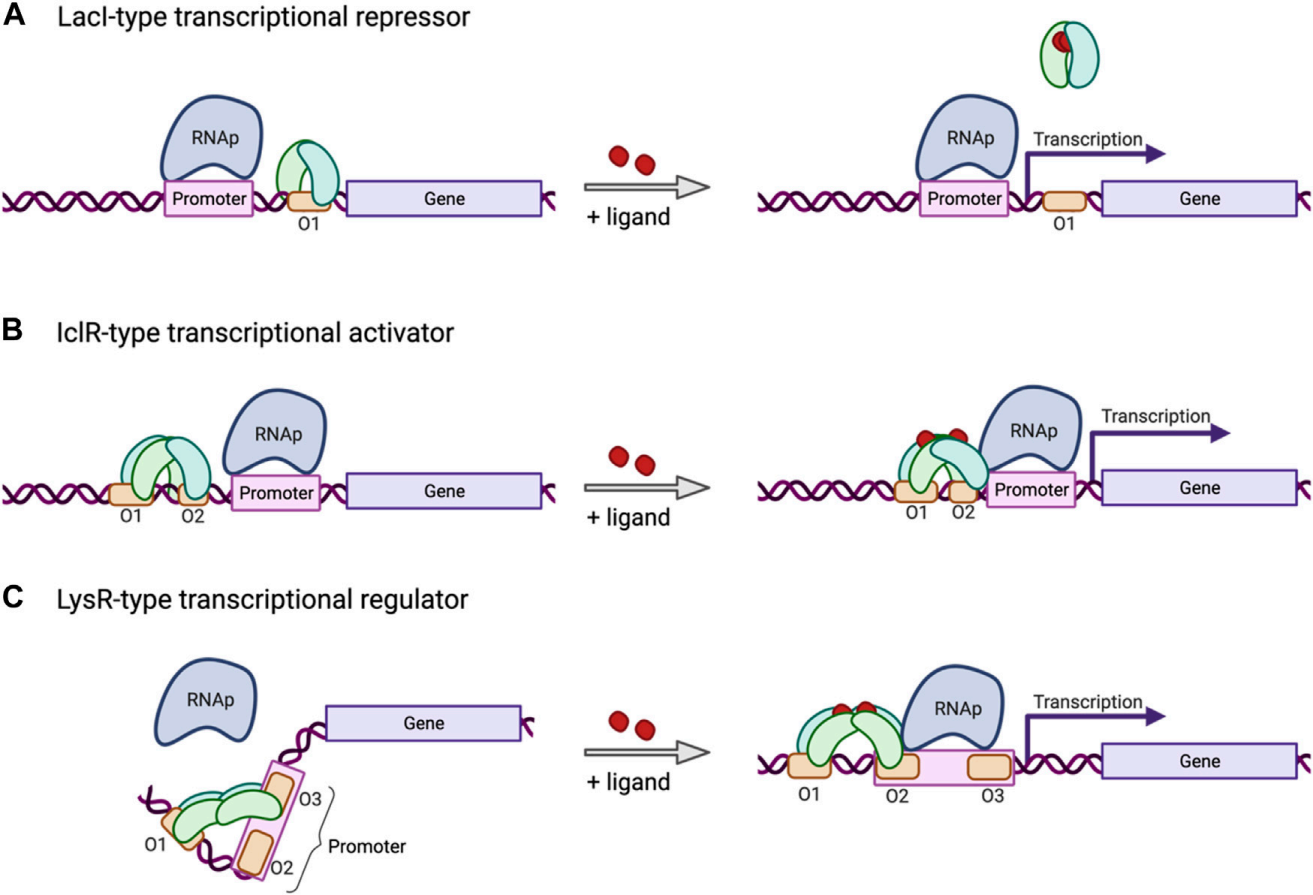
Mechanism of three common types of inducible transcriptional regulators. **(A)** The LacI-type transcriptional repressor binds an operator site located downstream from the promoter, or between the −10 and −35 sites, and blocks transcription, unless released by environmental factors, such as ligand binding. **(B)** The IclR-type transcriptional regulator induces transcription in response to environmental factors, such as ligand binding, inducing a conformational change in the tetramer bound to an operator site in proximity to the promoter. **(C)** The LysR-type transcriptional regulator (LTTR) binds operator sites 1 and 3 in its tetrameric apo form, bending the bound DNA and repressing transcription. In response to co-inducer binding, the LTTR tetramer will shift binding from operator site 3 to site 2, releasing the DNA bend, freeing up the promoter, and recruiting RNAp to allow transcription to be initiated. Created with BioRender.com.

The mechanism of promoter regulation can vary and is determined by the type of transcription factor that interacts with those promoters. The most common ones are transcriptional repressors that bind to specific regions in the proximity of the promoter and block transcription (Figure 2A). Repression can be released by the repressor reacting to changes in the environment, such as binding to a specific ligand. Certain other promoters are regulated by transcriptional activators (Figure 2B) that recruit transcription machinery in response to a certain change in the environment, such as accumulation or depletion of a certain metabolite or change in temperature. Another very common kind of transcriptional regulator in bacteria works as a repressor as well as an activator. The LysR-Type Transcriptional Regulator (LTTR) (Maddocks and Oyston, 2008) forms homotetramers with two arms each bearing a pair of DNA binding domains (Figure 2C). Typically, one of those arms remains anchored to an operator site in proximity to the promoter, regardless of whether the protein is in the apo form or bound to a co-inducer. The other arm typically shifts its position from a second to a third operator site in response to conformational changes brought about by activation, such as binding to a co-inducer. These conformational shifts can control gene expression by exposing or occluding the −35/−10 regions of the promoter, by altering DNA bending, or by direct interaction with the RNA polymerase complex. Mutations to specific regions on the operators that are differentially bound in the active or repressed state can alter the dynamics of the conformational switch. This strategy has been used to develop a biosensor for *cis,cis*-muconic acid in *P. putida* using an LTTR CatM from *A. baylyi* ADP1 (Bentley et al., 2020).

In addition, a meaningful biosensor application relies heavily on the dynamic range or the maximal fold change in response over the basal levels. Even if a transcription regulator is found that is responsive to the molecule of interest, the dynamic range may need to be altered through further promoter engineering, typically using the approaches described above, as well as protein engineering of the transcriptional regulator itself (Jha et al., 2016; Jha et al., 2018; Bentley et al., 2020) (described below).

### 1.2 Protein libraries

Proteins may need to be modified for a range of reasons, such as altering ligand binding, DNA binding, stability, activity or protein-protein interactions. When targeting which sequences to randomize, one may take either a random, semi-rational or fully rational approach. This is frequently determined by the amount of information available for the protein of interest, and impacts the library size to pursue. Typically, saturation mutagenesis is only used to fully randomize a few positions in the protein sequence simultaneously, otherwise, the number of possible combinations of variants quickly becomes impractical to build individually in the laboratory or test in a given timeline, even with state-of-the-art technologies.

Using rational targeted mutagenesis to alter the function, binding, or stability of a protein of interest is only a feasible approach if the structure and structure-function relationships of the protein are known. Ideally, the crystal structure of the protein is available to be pulled from a database such as PDB (Berman et al., 2000) or Uniprot (The UniProt Consortium Martin et al., 2021). Tertiary structures can also be predicted for proteins with a known sequence using computational approaches such as Rosetta (Rohl et al., 2004) or AlphaFold (Jumper et al., 2021). Further, the computational models facilitate docking of ligands in the putative binding sites, allowing for the visualization of residues to target for mutagenesis. *In silico* docking consisting of protein-protein or protein-ligand interactions (Lyskov and Gray, 2008; Lyskov et al., 2013) permits guided selection of amino acids involved in binding. Targeting only very relevant residues with computationally informed mutations, or conservative mutations such as neutral drift mutations (Lynch and Hill, 1986), can simplify library generation, reduce library size, and decrease noise and workload. *In silico* ligand docking in a comparative model to guide the design of a focused library was successfully used to engineer several biosensors for small molecules (Jha et al., 2015; Jha et al., 2016; Shin et al., 2022).

The ability to diversify multiple positions in a given sequence at once provides a significantly faster route to arriving at a combination of mutations providing appreciable alterations in phenotype. In addition, even imperfect predicted structures can be used to great effect by indicating promising residues to target for mutagenesis (Jha et al., 2016; Shin et al., 2022).

**FIGURE 3:**
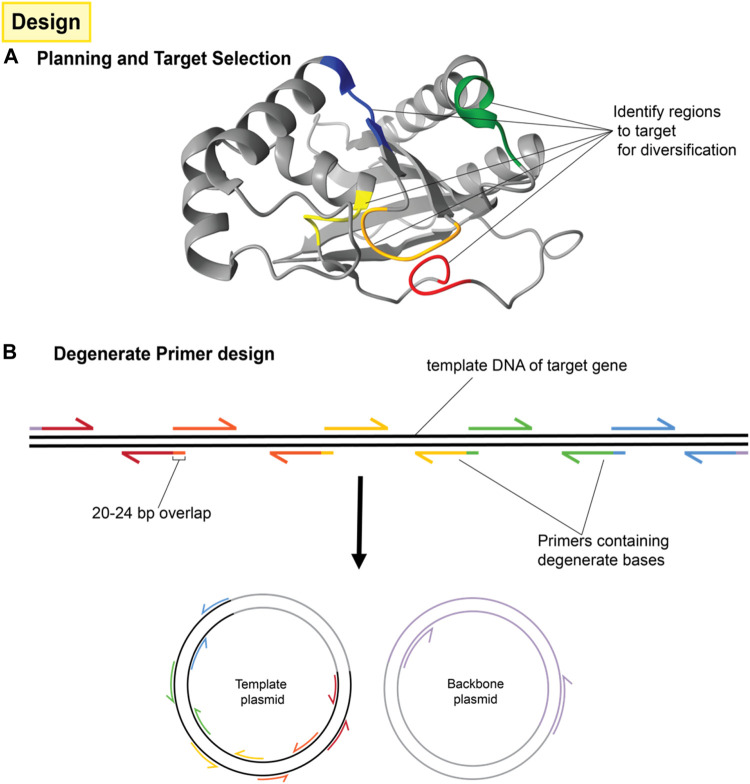
Design of protein mutation library and primers. **(A)** A number of residues are targeted for mutagenesis around the area of interest (typically a binding site or active site) but although those residues may be close to each other in the three dimensional structure, they may be far apart in the primary sequence. **(B)** The protein sequence is then separated into several fragments with the mutation sites located at one end of each fragment. This usually requires *n* + 1 fragments for *n* mutations unless one of the mutations is very near either end of the protein sequence. Each fragment will be generated by PCR reactions consisting of the template to be mutated and a pair of primers. One of those primers (the mutation primer) includes degenerate bases in the middle to effect mutation. The mutation primer must also include sufficient non-mutated bases on the 3′ end for binding and must include a 5′ overlap to the adjacent fragment. The other primer (the helper primer) does not need to contain degenerate bases and simply allows for the amplification of that fragment. Fragments are then combined by overlap extension PCR. Terminal primers include overlaps for cloning into the intended vector.

**FIGURE 4:**
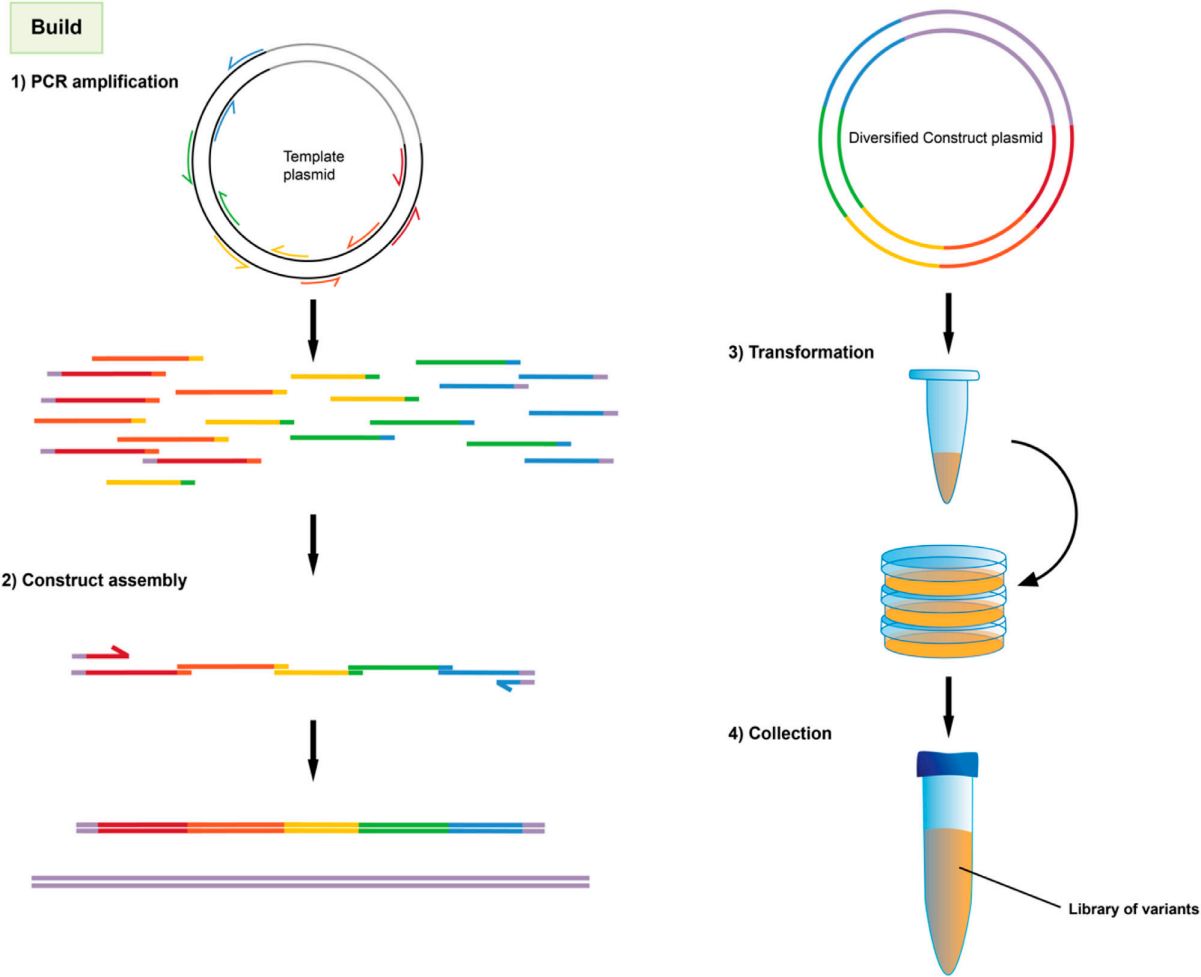
Overview of library construction method.

Once targeted positions in the genetic sequence have been identified, they are then mutagenized by PCR amplification and assembly using primers with incorporated degenerate codons, as shown in Figure 3. These degenerate codons have variation in the identity of the base at one or more positions such that the oligonucleotide pool contains unique sequences covering the range of mutations. Degenerate primers have long been used in the amplification and detection of panels of proteins, e.g., in virology (Li et al., 2012). When several degenerate primers targeting different regions of a gene are used for PCR fragments and assembly, the result is a combinatorial library consisting of all possible codon variations and combinations thereof as seen in Figure 4.

When designing the primers, it is helpful to refer to an amino acid substitution matrix, such as the Block Substitution Matrix 62 (BLOSUM62) table (Supplementary Table S1) (Henikoff and Henikoff, 1992). This 2-D matrix shows the log-odds score of finding two given amino acids in alignment, that is, comparing the occurrence of such an alignment to one that would be expected by random chance. A positive score indicates that this alignment is found with a higher frequency in nature than expected by chance, while a negative score indicates that this alignment is less frequent than expected. Practically, a positive score represents a statistically conservative substitution, while a negative score represents a non-conservative one. In the case of the BLOSUM62 matrix, the scores shown were determined based on sequences with an identity of 62% or less – making it useful for generating variants that are dissimilar to the starting sequence, but not entirely divergent (Eddy, 2004). Statistically conserved mutations form the basis of neutral drift mutations in an evolutionary trajectory, resulting in gain of functions (as in paralogs or orthologs).

The method described here lends itself well to generating large protein libraries, even based on limited initial information. Due to its capacity for screening the combinatorial effects of a broad range of mutations at once, it is possible to target several sites in a single protein for mutagenesis without significantly increasing the workload involved. This can be especially useful when constructing a new biosensor, where one may wish to target its capacity for ligand-binding, DNA-binding and multimerization all at once (Figure 3). This approach to protein engineering for biosensor development was successfully applied in the development of a protocatechuate biosensor in *P. putida* using an IclR transcription factor PcaU (Figure 2B) (Jha et al., 2018).

### 1.3 Applications of the method

The methodology outlined in this paper is broadly applicable to a range of objectives. Within the limitations outlined below (discussed in Section 1.4), we envision that this high-throughput approach using large libraries of diverse genetic variants, can be successfully employed in any project requiring random, or semi-rational design of pathways, proteins, and promoter variants. We have successfully applied this method to optimize promoter activity (Bentley et al., 2020; Pardo et al., 2020; Shin et al 2022), alter ligand binding (Jha et al., 2016; Shin et al 2022), reduce enzyme inhibition (Jha et al., 2019), construct biosensors (Jha et al., 2014; Jha et al., 2015) increase enzyme efficiency (Jha and Strauss, 2020), and improve thermostability and expression of an enzyme (Harrington et al., 2017), while taking advantage of the high throughput efficiency of flow cytometry.

### 1.4 Comparisons and limitations

#### 1.4.1 This method

The method described here introduces a variety of mutations at each mutation site by performing PCR-based, site directed mutagenesis, with primers containing degenerate bases at the desired mutation site. Multiple residues of the protein or positions in the promoter can be targeted simultaneously, with each targeted mutation region produced by a separate PCR reaction. Diversity is then further enhanced by the combinatorial assembly of those fragments by overlap extension PCR (Bryksin et al., 2013). The resulting gene or promoter library is then cloned into a vector by Gibson assembly (Gibson et al., 2009). While none of these three components (site directed mutation with degenerate primers, overlap extension PCR and Gibson assembly) are novel in isolation, their combination is rare within the mutation library field, despite offering superior flexibility, control of mutation bias, and ease of use when compared to the alternatives below.

#### 1.4.2 Alternatives to rational library design

The primary function of the mutation library is to create genetic diversity that can then be screened, tested, or even fed into some biological selection process. Genetic diversity can also be achieved by random mutagenesis promoted by chemical mutagens or radiation (Zhang et al., 2018). Given enough generations, mutations can also be accrued naturally, especially during adaptive laboratory evolution (ALE) (Zheng et al., 2021). The advantage of these alternative methods is that they do not require much up-front design work or cloning, although these techniques can be enhanced by selection methods, such as growth coupling (Godara and Kao, 2020) or biosensors with antibiotic resistance response (Yin et al., 2022).

The disadvantage of using these alternate methods lies in their more random nature. Without designing a bias towards potentially useful mutations, it is expected that a much larger percentage of the mutations will be deleterious, which requires screening or selecting from a larger sampling of the population. It is important to note, however, that ALE, random mutagenesis and mutation libraries are not mutually exclusive methods, but can complement each other. For instance, random mutagenesis, semi-rational libraries and even fully rational mutation libraries have been used to provide the genetic diversity for ALE to act upon (Arora et al., 2020).

#### 1.4.3 Alternative methods for targeted mutagenesis and library construction

There is a plethora of methods currently available to generate both random and targeted mutagenesis. Targeted mutagenesis approaches, such as site directed mutagenesis (SDM) and site-saturation mutagenesis (SSM) have proven to be extremely useful, however, the relatively low library size that can be achieved through these methods has restricted their application in directed evolution approaches (Sayous et al., 2020). Most recent technologies, such as sequence saturation mutagenesis (SeSaM), where a universal base is inserted along the target sequence, randomizing it at every single position (Wong et al., 2004), or casting error-prone PCR (cepPCR), where target DNA is fragmented and amplified using error-prone PCR (Yang et al., 2017), have increased achievable library sizes and mutational coverage. Yet, all of these methods required labor-intensive steps of cloning and transformation. The unprecedented drop in cost of DNA synthesis has allowed the generation of DNA libraries by high-throughput oligo synthesis (Kosuri and Church, 2014; Rocklin et al., 2017), enabling a complete saturation of small proteins. Still, the high price, compared to other techniques, makes this technique appropriate for only some specific applications.

After mutations have been generated using one of the above methods, a variety of cloning methods can be used for insertion into a replicating plasmid or genome. Traditional restriction/ligation methods have largely given way to PCR-based, recombination-based and CRISPR-based methods. The method described here uses Gibson assembly, which is versatile and familiar to many synthetic biology labs, but the protocol described herein could be easily adapted to use via megaprimer methods for insertion such as MEGAWHOP. The MEGAWHOP method traditionally consists of two PCR steps. The first step uses error-prone PCR for the generation of a set of megaprimers with random mutations in the target gene. The second step of PCR uses the megaprimers and the original plasmid as the template, resulting in a large random mutagenesis library (Miyazaki and Takenouchi, 2002; Miyazaki and Voigt, 2011). Compared to Gibson assembly, megaprimer methods have the advantage of requiring fewer enzymes and do not need a linearized backbone for insertion. Disadvantages include a higher incidence of mutations in the backbone as it is replicated by PCR. More advanced versions of the megaprimer method, such as QuickStep cloning (Jajesniak and Wong, 2015), have several advantages over earlier iterations, including exponential amplification of the whole plasmid and lower chances of self-annealing of the megaprimer at the 3′ ends.

Regarding chromosomally-targeted mutagenesis, homologous recombination (Recombineering) (Thomason et al., 2014) is the most commonly used technique. Recombineering requires specific single-stranded DNA annealing proteins that are highly specific and whose efficacy varies among different bacterial species, in addition to *in vitro* methods to generate sequence diversity.

CRISPR-based methods may also be used to mutate the genome directly. These methods exploit the sequence-specific mode of action of CRISPR, generally in combination with Cas9. For instance, CRISPR-enabled trackable genome engineering (CREATE) (Garst et al., 2017) is a method that makes use of large-scale oligonucleotide synthesis to generate a pool of 10^4^–10 (Salis et al., 2009) barcoded oligonucleotides, and then uses CRISPR to achieve mutagenesis within the genome. Additionally an *in vitro* CRISPR/Cas9-mediated mutagenic (ICM) system for construction of designer mutants in a PCR-free approach has been reported (She et al., 2018). In this method, CRISPR/Cas9 is used to cleave plasmid DNA at a target site, followed by T5 exonuclease digestion and annealing of primers containing the intended mutations. In both cases, CRISPR/Cas9 is used to cut the DNA strands, while the genetic diversity is achieved by synthetic DNA oligonucleotides containing the desired mutations. Both CRISPR and gene synthesis methods can eliminate bias when creating DNA libraries, however, PCR methods, such as the one presented here, remain the most widely applied, due to their low cost and ease of use (Table 1). Furthermore, the method described here allows for control of bias mutations, since the design of degenerate primers will allow saturation or partial saturation mutagenesis. This method can be used in non-model organisms, helps achieve both combinatorial and targeted mutagenesis, can be performed using relatively simple and widely used molecular biology techniques, and can also be employed in combination with other mutagenesis methods described in Table 1.

**TABLE 1:** Comparison of methods used for generation of mutation libraries.

Name (Acronym)	Description	Advantages	Disadvantages
Site-directed mutagenesis (SDM) (Sayous et al., 2020)	A specific mutation is introduced in a gene by primers. One or two adjacent amino acids can be mutated per primer	Focused and very specific. Ideal for targeting single bases for mutation. Useful for the creation of small libraries (8.5 h–2 days for 10–100 variants) (Bachman, 2013). High accuracy (>80%) if using high-fidelity DNA polymerase (Alejaldre et al., 2021)	Each target mutation requires an individual mutagenesis reaction, thus cost and time limit the achievable library size. Only useful if the target protein is already very well characterized. Efficiency relies on the availability of suitable cloning systems (Alejaldre et al., 2021)
Site-saturation mutagenesis (SSM) (Sayous et al., 2020)	One amino acid is mutated to the other 19 amino acids by whole-plasmid PCR with premers consisting of degenerate codons (Kretz et al., 2004), achieving all possible mutations for a single position	Larger genetic diversity than SDM (Siloto and Weselake, 2012). Reduces mutational bias	Workload similar to SDM, requires individual mutagenesis reaction and transformation for each target residue, not combinatorial
Sequence saturation mutagenesis (SeSaM) (Wong et al., 2004)	DNA fragments with a random spread of sizes are generated and elongated at the 3′ end using a universal base. The universal base is then randomly replaced with standard nucleotides during PCR, generating a randomized sequence	Large diversity of mutants, can theoretically achieve full randomization of a desired sequence	Moderately labor-intensive (2–3 days to generate a library), involved multi-step PCRs, universal base introduces mutation bias, interval of mutations depends on fragmentation method used, not combinatorial, introduces stop codon at a frequency of 0.03
Casting error-prone PCR (cepPCR) (Yang et al., 2017)	Target DNA is amplified into 100-200 bp fragments. Each fragment is subjected to high-rate error-prone PCR. Mutant fragments are then used as megaprimers (see MEGAWHOP) to generate a library of mutants for each fragment	Reduces redundancy of mutations, achieves higher mutation rate than traditional error prone PCR.	Moderately labor-intensive (1–2 days to generate libraries), mutation bias, only achieves a subset (<40%) of potential beneficial mutations. The multiple resulting libraries may need to be screened individually, limiting this method to shorter sequences
Megaprimer PCR of whole plasmid (MEGAWHOP) (Miyazaki, 2011)	A megaprimer carrying desired mutations is hybridized to the template DNA for whole plasmid PCR. The original template is then degraded by *DpnI* digestion	Produces variants with a combination of mutations. Can be combined with cepPCR or degenerate nucleotides, no need for ligation or assembly	Synthesizing megaprimers may carry high costs, In-house generated megaprimers have the same shortcomings as the method used to generate them (i.e., epPCR or cepPCR). Higher chance of introducing mutations in the backbone
Library DNA synthesis (Kosuri and Church, 2014; Rocklin et al., 2017)	DNA libraries are created by high-throughput oligo synthesis to user specifications	Eliminates mutation bias, large library size (>10^6^) and custom-fit. Reduced workload for user	High cost ($0.2-0.5 per bp for large genes). Potentially longer lead times to receive library
Homologous recombination (Recombineering) (Thomason et al., 2014)	A previously made DNA fragment is inserted in the genome by homologous recombination	Mutants can be introduced in the genomic DNA, improving stability and eliminating the need for reporters	Needs highly specific DNA annealing proteins, and optimization
CRISPR-enabled trackable genome engineering (CREATE) (Garst et al., 2017)	CRISPR/Cas9 is used to cut genomic DNA, and barcoded oligonucleotides are inserted in the desired position	Genome editing at multiple loci, mutations can be tracked. Allows >50,000 genome wide mutations (Tarasava et al., 2018). Used in *E. coli* and *S. cerevisiae*. Off-target mutations are expected to be low, no mutation bias, relatively easy to perform	Requires potentially expensive barcoded oligo synthesis (ex: ∼$5000 for a 10^5^-variant library of 50bp oligos (Garst et al., 2017)). Low efficiency (>70%), requires development of Cas system, has not been demonstrated in mutagenesis of single, specific enzyme
*In vitro* CRISPR/Cas9-mediated mutagenic (ICM) (She et al., 2018)	CRISPR/Cas9 is used to cut plasmid DNA, and then mutation carrying oligonucleotides are inserted in the desired position	Off-target mutations are expected to be low, no mutation bias, no need for restriction enzyme digestion, PCR, restriction sites or plasmid size limit	As with CREATE, high cost due to the need for high-throughput oligo synthesis
This method	Combines overlap extension PCR and saturation or partial saturation mutagenesis with degenerate primers	Allows for rapid in-house creation of large, combinatorial variant libraries (≥10^6^), bias mutations can be controlled, low cost (e.g., only a dozen 18–50 bp primers for 5 mutation sites spread over a gene or fewer primers if some mutations are near enough to each other such that they may be covered by a single primer)	Moderately labor intensive (2–3 days for library construction)

#### 1.4.4 Limitations of this method

The method we present here is capable of quickly generating large libraries of genetic variants and quickly screening them for desired phenotypes. However, the construction of the library relies on degenerate primers. These are produced in such a way as to control the ratio of nucleobases at a given location, but the different sequences may show differences in annealing during PCR resulting in a degree of bias. Ensuring sufficient 3′ complementarity after the mutation site mitigates this effect, but the exact ratio in the final library is not always known. Thus, this protocol includes a verification step of either sequencing a random selection of clones isolated from the library (see Figure 6A), or sequencing DNA extracted from the whole library (see Figure 6B), for quality assurance. This provides reasonable certainty that the desired mutations were achieved, and that the library contains a sufficiently broad range of variants to be of interest for screening.

This method is additionally limited by availability of knowledge about the targeted protein or promoter (see Section 1.2), as well as by the expertise of prospective user regarding FACS. Additionally, if looking to build a biosensor/binder for a molecule that is readily metabolized or exported, then it may be challenging to achieve an intracellular concentration sufficient for screening by FACS, and metabolic engineering to disable one or more metabolic pathways may be required.

### 1.5 Expertise needed

In order to successfully apply this protocol, the prospective user will need experience with basic molecular biology methodologies, including: polymerase chain reaction, agarose gel electrophoresis, gel extraction, bacterial transformation, and bacterial culture, as well as a fundamental understanding of molar ratios and calculations. Experience using FACS is required. Additionally, familiarity with primer design and gene editing software is necessary. Sequences for the targeted gene or promoter need to be known or obtainable.

When targeting a gene for diversification, it is helpful to have an understanding of the protein encoded therein, and its structure-function relationship. Familiarity with Rosetta (Rohl et al., 2004) or AlphaFold (Jumper et al., 2021) and its protein folding and ligand docking functionality is beneficial for rational protein diversification approaches. Similarly, for promoter engineering, an understanding of promotor features and transcription factor mechanisms assists with targeting specific regions for mutagenesis (Browning and Busby, 2004; Saecker et al., 2011).

## 2 Materials

### 2.1 Reagents

• Oligonucleotides can be purchased from Eurofins or other vendors. Oligonucleotides are dissolved in ultrapure water to a concentration of 50 μM
• UltraPure™ DNase/RNase-Free Distilled Water (Invitrogen, cat. no. 10977015)
• Deoxynucleotide (dNTP) solution mix (New England Biolabs, cat. no. N0447L)
• High-fidelity DNA polymerase (e.g., New England Biolabs, Phusion DNA polymerase, cat. no. M0531L, Q5 DNA polymerase Q5^®^ High-Fidelity 2X Master Mix, cat. no. M0492L)
• Dimethyl sulfoxide (DMSO) Fisher BioReagents, cat. no. BP231-100
• Agarose (e.g., Invitrogen, UltraPure™ Agarose, cat. no. 16500100)
• Gel Loading Dye, Purple (6X), no SDS, cat. no. B7025S
• DNA ladder (e.g., New England Biolabs, 1 kb DNA Ladder, cat. no. N3232L, 100 bp DNA Ladder, cat. no. N3231L)
• GelRed^®^ Nucleic Acid Stain 10,000X Water (Sigma, cat. no. **SCT123**)
• TAE Buffer (Tris-acetate-EDTA) (50X) (Thermo Fisher Scientific, cat. no. B49)
• QIAquick Gel Extraction Kit (QIAGEN, cat. no. 28706X4)
• QIAquick PCR Purification Kit (QIAGEN, cat. no. 28104)
• MinElute Reaction Cleanup Kit (QIAGEN, cat. no. 28204)
• Restriction enzymes and 10X reaction buffer
• T4 DNA ligase (New England Biolabs, cat. no. M0202L)
• Antarctic Phosphatase (New England Biolabs, cat. no. M0289S)
• High-efficiency bacterial competent cells (e.g., Thermo Fisher Scientific, MAX Efficiency™ DH5α, cat. no. 18258012)
• Assembled plasmid DNA library, user supplied.
• SOC Outgrowth Medium (New England Biolabs, cat. no. B9020S,
• Bacterial growth and selection medium (liquid and agar) e.g., Luria Broth Base (Thermo Fisher Scientific, Miller’s LB Broth Base, cat. no. 12795027
• Antibiotics (e.g., kanamycin sulfate, Thermo Scientific, cat. no. 11815024)
• Phosphate buffered saline (PBS) (G-Biosciences, cat. no. 786-027)

### 2.2 Equipment

• Incubators at appropriate temperature and agitation
• Thermocycler (e.g., Applied Biosystems 2720 Thermal Cycler)
• Gel electrophoresis system (e.g., Thermo Fisher Scientific, Owl™ EasyCast™ B1A Mini Gel Electrophoresis Systems, cat. no. B1A-BP)
• ChemiDoc Imaging System (Bio-Rad)
• Cell scrapers
• Tube rotator
• Flow cytometer (FACSAria III flow cytometer) capable of cell sorting based on fluorescence

### 2.3 Standard laboratory consumables

• PCR tubes
• 1.5 and 2 mL Eppendorf tubes
• 14 mL culture tubes
• Petri dishes

### 2.4 Software

• SnapGene or similar
• Protein modeling software such as ChimeraX or PyMOL
• Rosetta or AlphaFold

## 3 Procedure

### 3.1 Overview

This protocol can be divided into three main components: Library Design, Library Construction, and Library Screening analogous to the Design, Build, Test framework of engineering principles for synthetic biology (Peccoud, 2016; Opgenorth et al., 2019). The three main components can be further divided into individual steps as described in Figure 1. The principals associated with Library Design were discussed in Sections 1.1, 1.2 above. The protocols for Library Construction and Library Screening are detailed below.

In total, this method will take approximately 3 weeks from initial library design to isolating and characterizing individual clones, for a reasonable library size of 10^5^–10^6^, with the workload for each day itemized below. Depending on the library size, a smaller or larger workload can be expected for smaller or larger libraries respectively.

Day 1: PCR round 1 to generate fragments. Agarose Gel electrophoresis and gel extraction.

Day 2: PCR round 2 to assemble, Agarose Gel Electrophoresis and gel extraction,

Day 3: Transformation (include main plates and transformant estimation plates).

Day 4: Plate scraping (and colony counting), direct use or glycerol stock, extractions for sequencing, inoculation of liquid culture.

Day 5: Re-inoculation and induction.

Day 6: First round of analysis by flow cytometry and sorting, followed by outgrowth (NOTE: In order to collect rare clones, in the first round of sorting it is recommended to collect the top 5% of the library).

Day 6: Glycerol stocks, liquid culture of round 1 populations.

Day 7: Re-inoculation of round 1 populations, induction.

Day 8: Second round of analysis and sorting, outgrowth (NOTE: A negative sorting is recommended to eliminate constitutive performers, especially observed when engineering regulatory proteins and promoters).

Day 9: Glycerol stocks and liquid culture of round 2 populations.

Day 10: Re-inoculation and induction.

Day 11: Third Round of screening and sorting, outgrowth (NOTE: Increased stringency in sorting, i.e., collecting only the top 1%–2% of population is recommended).

Day 12: Glycerol stocks and liquid culture of round 3 populations.

Day 13: Re-inoculation and induction.

Day 14: Fourth round of screening and sorting, plating with appropriate antibiotic selection (NOTE: Stringently collecting only the top 1% of performers is recommended).

Day 15: Picking 24-48 colonies of individual clones.

Day 16–19: Test individual clones at different conditions in order to characterize properties.

### 3.2 Primer design and construction

The following instructions detail the production of a hypothetical mutation library, wherein two distant regions of a promoter or protein are diversified. Mutation sites can range in size from a single base pair up to any stretch of the sequence reasonably covered by a single PCR primer after factoring in the 3′ overlap needed for the initial PCR and the 5′ overlap needed for overlap extension PCR described below.

#### 3.2.1 PCR based mutagenesis: (1 day)

1. Primers are designed according to standard site-directed mutagenesis and overlap extension PCR strategies (Ho et al., 1989; Heckman and Pease, 2007). For our example of two mutation sites, a total of six primers are needed; one degenerate primer at each mutation site with 20 base pair overlaps that extend into adjacent fragments, one non-variable primer for each mutation site to serve as the reverse primer for that fragment and finally, two primers to flank the entire region. This divides the promoter into three fragments separated by mutation sites. Figure 3B shows the same concept, but for four mutation sites. NOTE: It is sometimes necessary to use more degenerate primers at each mutations site. For instance, if the library is designed to include three possible codons at a given amino acid position; GCA, CAG, and GAA (for alanine, glutamine and glutamic acid, respectively), then it would *not* be appropriate to use a single primer containing the degenerate bases SMR (See Supplementary Table S2), because these could also combine to code for proline. Instead, two different primers containing GMA or CAG could be used and mixed 2:1 in the PCR reaction for all three amino acids to be equally represented.
2. PCR is performed for each fragment separately. For primers with degenerate bases, annealing temperatures should be lowered to accommodate the mutation variant with the least stable hybridization to the template. Follow suggested thermocycler settings for whichever high-fidelity polymerase is used.
3. The entire PCR product is then run on an agarose gel and the correct sized bands are excised.
4. Extract DNA from the gel excisions using commercially available gel extraction kits (e.g., QiaQuick gel extraction kit)

#### 3.2.2 Construct assembly (1 day)

5. PCR fragments are then combined via overlap extension PCR. In the first stage of overlap PCR, the fragments (at equimolar ratio) and PCR reagents/enzymes are allowed to react for 8 cycles with an extension time sufficient to copy the largest fragment. Then, primers flanking the entire promoter region are introduced and 25 more cycles are performed with an extension time sufficient for the entire region. Subsequent purification of the PCR product with commercial kits assists the next step (i.e., QiaGen PCR Cleanup Kit).
6. The mutated variant library is then assembled into a linearized vector using Gibson assembly (Gibson et al., 2009; Thomas et al., 2015) or restriction digestion/ligation. Restriction sites may be introduced into the primers used to flank the promoter in step 5.

#### 3.2.3 Transformation (1 day)

7. The assembled plasmid library is transformed into a suitable competent bacterial strain, Transformation may be performed by heat shock or electroporation. Depending on the known or expected transformation efficiency, care must be taken to perform sufficient transformations to achieve appropriate coverage of the library. Commonly one would aim to obtain at least 4-fold coverage e.g., generating ≥1 million transformants for a library with a theoretical diversity of 250,000. This is to ensure that the maximum number of variants is represented in the bacteria.

After transformation and recovery, it is advisable to plate approximately 20 µL of the recovered bacteria on an agar plate containing the appropriate medium and selective antibiotic. This is to estimate transformation efficiency and therefore the final degree of coverage of the library that was achieved (i.e., quantification plate). The remaining recovered bacteria are gently spun down (4,000 rpm, 4–5 min), and the majority of the supernatant recovery medium is removed to allow for plating of the entire volume of transformants. Once plated, the transformants are incubated overnight at a suitable temperature to form colonies.

#### 3.2.4 Collection and stocks (1 day)

8. Using the quantification plate from step 7, the number of transformants achieved is estimated. If the desired coverage is achieved, the whole library can be pooled by adding a small amount of liquid medium to the plates (typically 1 mL), and then gently scraping the colonies to collect, using a cell scraper. Repeat the addition of liquid media, scraping and then pool the resulting cell suspensions in a polypropylene tube (15–50 mL, depending on resulting volume), sealed tightly, and then rotated for at least 1h to ensure proper mixing of the collected library.
9. Glycerol stocks of the collected library are prepared by adding glycerol to aliquots of the library to a final concentration of 20% glycerol, taking note of final OD of the resulting stock. These stocks are suitable for long-term storage in ultracold freezers (−80°C). Note that when reviving culture from stocks it is essential to use sufficient inoculum to achieve full coverage of the library i.e., inoculate fresh culture with a number of cells at least 10-fold greater than the total number of variants in the library.

#### 3.2.5 Verification (2–5 days)

10. In order to verify the integrity and diversity of the library, plasmid DNA is isolated from individual clones (e.g., picked from the quantification plate in step 7 and grown up overnight) or from a small volume of the collected library, using any desired plasmid DNA extraction method (i.e., QiaGen Miniprep Kit). The isolated DNA, along with an appropriate primer, is sent for sequencing by one’s preferred provider (in-house, Twist, Eurofins, etc.). Depending on usual shipping and processing times, it may take several days for the sequencing data to become available. See Figures 6A, B for an example of expected results.

### 3.3 Library Screening

#### 3.3.1 Two rounds of positive selection (3–5 days)

**FIGURE 5:**
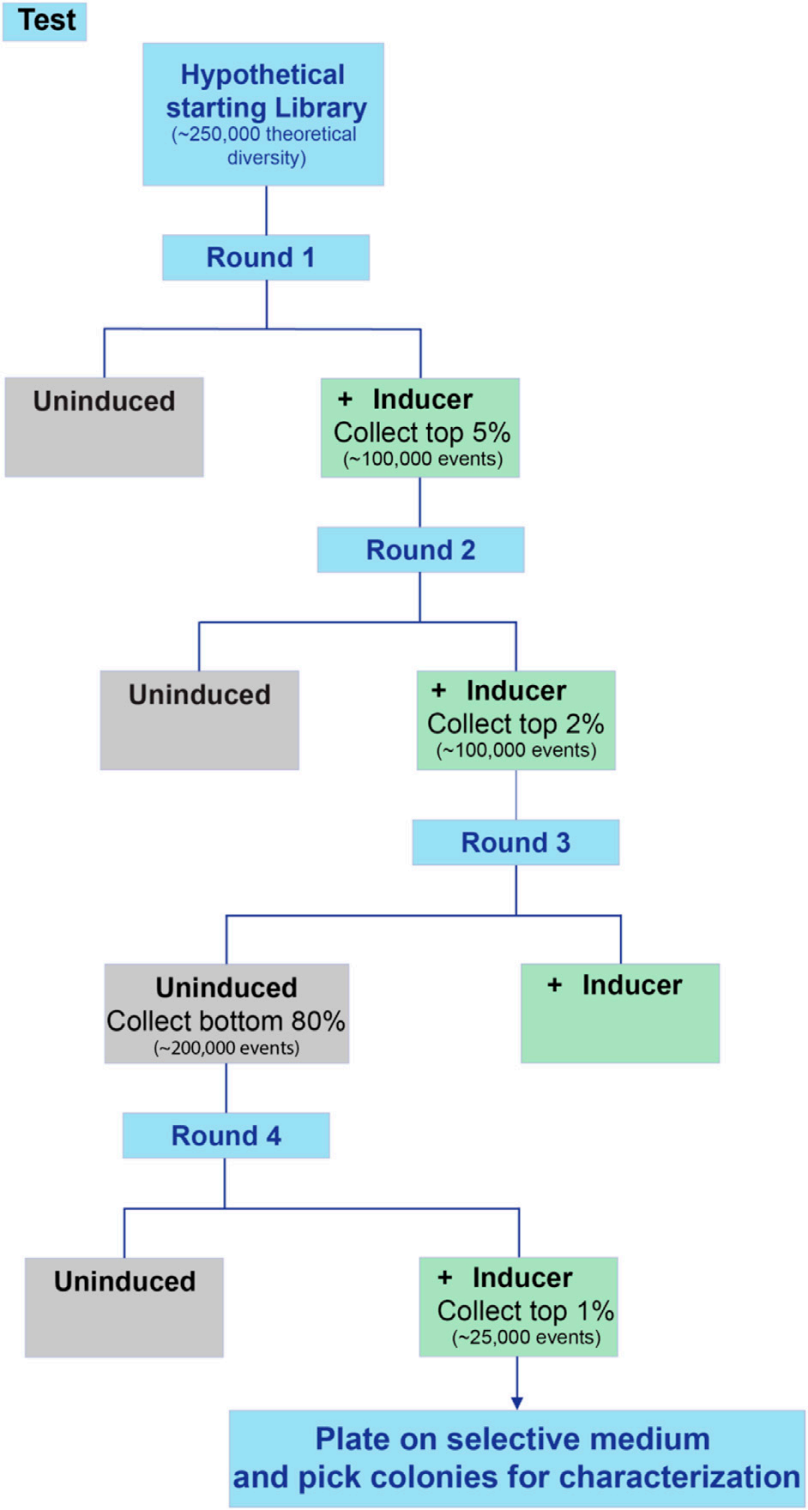
Workflow for screening an inducible promoter library. When screening inducible promoters, it is advisable to include several rounds of positive selection (high expression when induced) as well as at least one round of negative selection (low background expression when uninduced). This example includes two rounds of positive selection, one round of negative selection, and a final round of positive selection.

11. The library should be screened using media and other growth conditions mirroring the application and desired effect of the mutations. This method assumes testing of cells at mid-log growth phase. Prepare an overnight culture in 3 mL of selective liquid media, using either the scraped cell suspension from step 8 (if available) or the glycerol stock of the library. Use a sufficiently large volume of inoculum to achieve library coverage (see Step 9).
12. Use the overnight culture to inoculate a fresh 3 mL culture to an initial OD that is about 1/10th of the strain’s stationary phase OD in that media. Incubate the cells with frequent OD monitoring until they reach approximately 50% of stationary OD (mid-log phase). If induction is required, induce at mid-log phase and allow more time for the induced process to proceed.
13. Dilute a small portion of the cells in 1X phosphate buffered saline to achieve a cell density of approximately 10^7^cells/mL for flow cytometry. Required dilution may vary depending on the requirement of the instrument used, since efficient sorting requires an event rate well below the recommended maximum event rate for any given flow cytometer.
14. Select the top five percent best performing cells based on biosensor response and sort into fresh (selective) media. Grow the sorted cells overnight to recover.
15. Repeat steps 12-14, this time collecting the top two percent only.
16. If induction was used, or the library is for a new biosensor or promoter to be optimized, it may be necessary to screen for negative fluorescence in the uninduced state i.e., select a non-fluorescent subset of the uninduced population. This will eliminate constitutively active variants, facilitating isolation of true inducible variants. If deemed necessary, repeat steps 12-14 without induction and collect the bottom 80% in terms of fluorescence.
17. Alternate between induced selection and uninduced selection until the desired phenotype is reached or no further improvement is observed between rounds of sorting. An example screening flowchart for an inducible process is shown in Figure 5. Note that sorts with a wider selection window in terms of percentage can include more cells collected, but that sorts with narrow windows have fewer cells collected in the interest of time.
18. Plate cells from the last sort onto selective media and pick individual colonies for characterization.

## 4 Expected results

**FIGURE 6:**
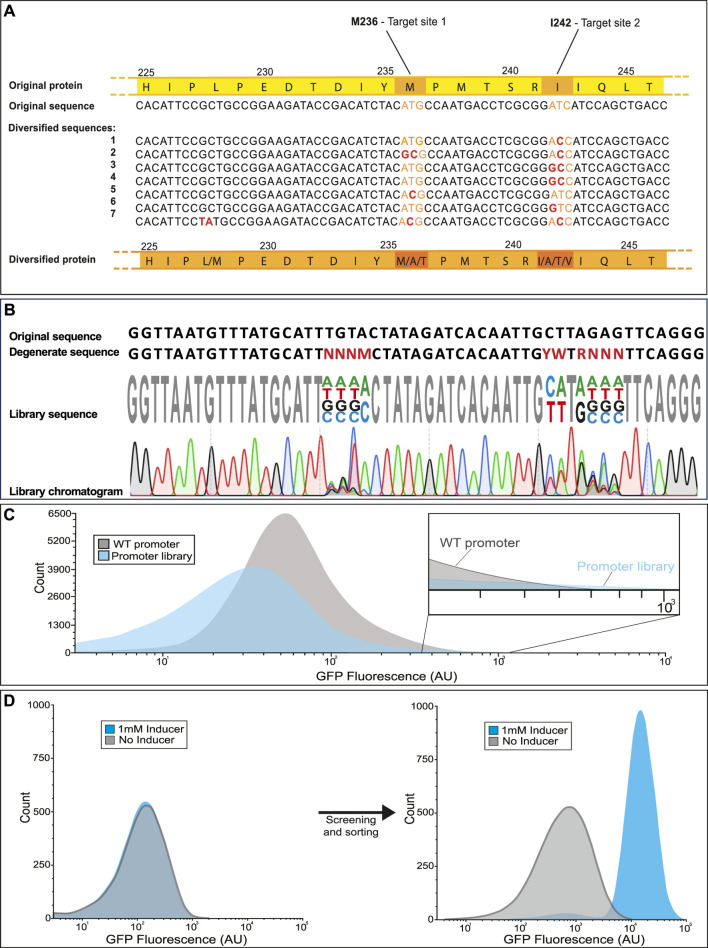
Expected results for library validation in various applications. The library is first sequenced to ensure that each mutation site exhibits the expected variability. This can be performed in two different ways; **(A)** by sequencing several isolates and aligning them for comparison or **(B)** by sequencing the mixed population and observing overlapping peaks in the chromatogram. **(C)** Ideally the library exhibits a broader distribution of phenotype then that of the parent strain. It is acceptable if many of the mutations are deleterious as long as the rare strains with improved performance can be selected from the library (See inset). **(D)** After selection, high performing mutants can be compared to the wild type by cytometry. An example for isolating a novel biosensor by diversifying a wild type transcription factor is shown. The wild type transcription factor CatM (right) shows no response to the added inducer, while the variant isolated after several rounds of screening (left) displays a >40-fold increase in fluorescence in response to the inducer (Bentley et al., 2020).

During construction of the library, there will be multiple tests performed at intermediate phases to ensure the components are being generated and assembled correctly. During the initial PCR steps, amplification products are observed on agarose gel and can be checked for the correct size. After the gene is inserted into a vector by Gibson assembly and transduced into a host, a subset of the transformation culture is diluted and used for counting plates (quantification plate) which have the additional benefit of providing isolated colonies from which to test the diversity of the library at each mutation site prior to employing high throughput screening or selection. Before screening, a check may be included to test for mutation diversity (Figure 6). One example, shown in Figure 6A involves selecting several random isolates from a library which are sequenced (Sanger method) at the mutation sites. Given the degenerate bases provided, there are three possible amino acids (including the wild type) for the inducer binding residue M236 and four possible amino acids for the dimer interface I242. For each mutation site, every possible amino acid substitution was observed in the seven isolates. Alternatively, the library may be sequenced directly, without isolating individual strains. In this approach, degenerate bases should produce multiple peaks of different fluorophores on the sequencing chromatogram depending on the rate of base-pair substitutions (Figure 6B).

Based on the goals set forth for the library design, the library is expected to exhibit a broad distribution of phenotype in addition to genotype. In the case of libraries whose performance can be tested and screened by fluorescence, the phenotypic range can be measured on a flow cytometer. In the example histogram provided (Figure 6C), the majority of mutation combinations for a promoter library are deleterious, causing the average fluorescence to go down. The strength of the library lies in the rare mutations with gain of function or improved performance, evident in the high fluorescence tail of the library population which extends slightly beyond the high fluorescence region of the wild type population. By selecting cells from those regions of the library histogram that perform better than the wild type, it is possible to isolate variants with markedly improved function. One example is a cis,cis-muconanic acid biosensor isolated from a biosensor library (Figure 6D). While the wild-type transcription factor (Figure 6D left) showed no visible response to the inducer, the variant isolated from the library (Figure 6D right) responded remarkably well to induction, with only a minor shift in background fluorescence compared to the wild-type.

## 5 Conclusion

Current methods designed to identify gain-of-function mutations for proteins or promoters in microbes are limited by workload, time, and costs. The methodology described here eliminates the bottleneck of testing individual variants of genes and promoters by presenting a protocol for assaying the combinatorial effect of several mutations at once, drastically reducing the time and money required to obtain genetic variants with desirable qualities. The efficacy of the method described here is illustrated by several of our original research papers (Jha et al., 2014; Jha et al., 2016; Jha et al., 2018; Shin et al., 2022), which have successfully employed this approach. The versatility and usefulness of this high-throughput screening method applied to large genetic libraries is therefore evidenced in published literature. While the individual components of this workflow (overlap extension PCR, degenerate primers, FACS, etc.) may seem commonplace, we have not encountered any other methodology that combines them in the fashion outlined in this paper.

Due to the high-throughput nature of the method detailed here, the number of genetic variants that can be screened for desired gain-of-function behavior is not limited by the time or manpower available. Instead, millions of variants can conveniently be screened for desirable phenotypes in a single sample tube. Variants that perform well are isolated by FACS and screened further in subsequent rounds, while poor performers are easily discarded.

## Data Availability

The original contributions presented in the study are included in the article/Supplementary Material, further inquiries can be directed to the corresponding author.
