# Supplementary material for: Targeted mutagenesis and high-throughput screening of diversified gene and promoter libraries for isolating gain-of-function mutations
Source: Front Bioeng Biotechnol. 2023 Jul 17;11:1202388. doi: 10.3389/fbioe.2023.1202388 (PMC10400447; doi:10.3389/fbioe.2023.1202388)
Supplement: Supplementary file 1 [file Table1.docx]

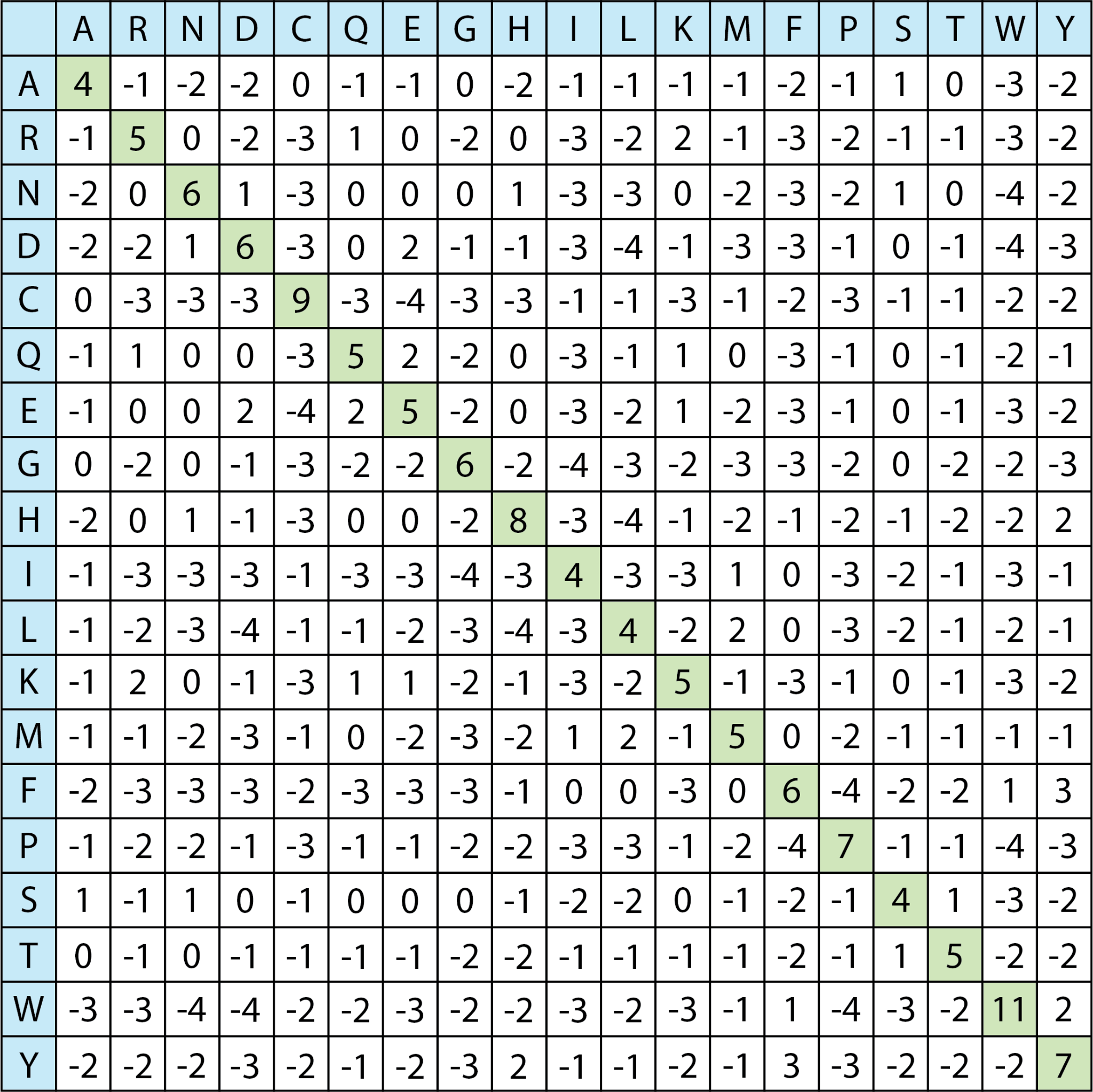


Table S1: **Blo**ck **Su**bstitution **M**atrix 62 (BLOSUM62) This 2-D matrix shows the log-odds score of finding two given amino acids in alignment relative to the odds expected by random chance. Higher scores represent a statistically conservative substitution while a negative score represents a non-conservative one.
