# Supplementary material for: Targeted mutagenesis and high-throughput screening of diversified gene and promoter libraries for isolating gain-of-function mutations
Source: Front Bioeng Biotechnol. 2023 Jul 17;11:1202388. doi: 10.3389/fbioe.2023.1202388 (PMC10400447; doi:10.3389/fbioe.2023.1202388)
Supplement: Supplementary file 2 [file Table2.docx]

| **Code** | **Nucleotides** | **Complement** |
| --- | --- | --- |
| **A** | A | T |
| **G** | G | C |
| **C** | C | G |
| **T** | T | A |
| **R** | A or G (purine) | Y (pyrimidine) |
| **Y** | C or T (pyrimidine) | R (purine) |
| **S** | C or G (strong pairing) | S (strong) |
| **W** | A or T (weak pairing) | W (weak) |
| **K** | G or T (keto) | M (amino) |
| **M** | A or C (amino) | K (keto) |
| **B** | C, G or T (not A) | V (not T) |
| **V** | A, C or G (not T) | B (not A) |
| **D** | A, G or T (not C) | H (not G) |
| **H** | A, C or T (not G) | D (not C) |
| **-** | gap | - |

Table S2: IUPAC codes for degenerate nucleotides. Oligonucleotide synthesis vendors that are capable of including degenerate bases typically use IUPAC notation.
